# Draft Genome Sequence of Environmental *Vibrio cholerae* 2012EL-1759 with Similarities to the *V. cholerae* O1 Classical Biotype

**DOI:** 10.1128/genomeA.00617-14

**Published:** 2014-07-10

**Authors:** Lee S. Katz, Maryann Turnsek, Amy Kahler, Vincent R. Hill, E. Fidelma Boyd, Cheryl L. Tarr

**Affiliations:** a Division of Foodborne, Waterborne, and Environmental Diseases, Centers for Disease Control and Prevention, Atlanta, Georgia, USA; b Department of Biological Sciences, University of Delaware, Newark, Delaware, USA

## Abstract

*Vibrio cholerae* 2012EL-1759 is an environmental isolate from Haiti that was recovered in 2012 during a cholera outbreak. The genomic backbone is similar to that of the prototypical *V. cholerae* O1 classical biotype strain O395, and it carries the *Vibrio* pathogenicity islands (VPI-1 and VPI-2) and a cholera toxin (CTX) prephage.

## GENOME ANNOUNCEMENT

Cholera toxin-producing *Vibrio cholerae* O1 was first detected in Haiti in October 2010, and both the epidemiological and genomic data are consistent with the notion of a single exogenous source for the ongoing outbreak (1–3). Because the ingestion of contaminated drinking water is the primary mode of cholera transmission, the Centers for Disease Control and Prevention initiated the surveillance of surface waters in Haiti to assess the risk of exposure from water and seafood (4). In addition to recovering the toxigenic epidemic strain, nontoxigenic non-O1/O139 *V. cholerae* isolates that were positive by PCR for *tcpA* were also recovered from Haitian waters (Kahler, Haley, Chen, Mull, Tarr, Turnsek, Katz, Humphrys, Freeman, Boncy, Colwell, Huq, and Hill, unpublished data). Because *tcpA* is part of the *tcp* gene cluster that encodes the toxin coregulated pilus (TCP), a major colonization factor and the receptor for the cholera toxin phage (CTXΦ) (5), we characterized a representative isolate by whole-genome sequencing.

Whole-genome sequencing was performed using the Illumina MiSeq, according to the manufacturer’s protocols, to generate 2 × 150 bp reads, which were assembled using SPAdes version 3.0 and scaffolded using SOPRA version 1.4.6 to give an assembly size of 3.98 Mb, with an *N*_50_ of 363 kb (6, 7). The gene predictions and annotations were performed using the Computational Genomics Pipeline (CG-Pipeline) version 0.4 and RAST (8, 9), which predicted and annotated 3,533 coding genes. The G+C content is 47.5%.

A phylogeny was constructed based on core genes, and 2012EL-1759 was found to be most closely related to the *V. cholerae* O1 classical biotype strain O395, with only 2,285 single-nucleotide polymorphism (SNP) differences across the core genome. The annotated genome includes the *Vibrio* pathogenicity island (VPI), a 40-kb region that encompasses the *tcp* gene cluster (10). The presence of a complete VPI suggests that the strain can express the TCP. We used MAUVE (11) to show that the VPI region, including *tcpA*, is more similar to the VPI of the classical biotype than to the VPI of the El Tor biotype, and the *tcpA* gene differs from the classical allele at two nucleotide sites. The cholera toxin genes (*ctxAB*) were not detected by PCR, nor were they found in the genome assembly or in the Illumina reads. However, core CTXΦ genes (*cep*, *orfU*, *ace*, and *zot*) and an RS1 element (*rstR*^*Calcutta*^, *rstA*, and *rstB*) appeared to be in the *dif* site on chromosome 1. There was no evidence of core phage genes present in the *dif* site on chromosome 2, but *rst* genes were present.

Boyd, Heilpern, and Waldor (12) described a model of *ctxAB* cassette acquisition; we suggest that the genome represented by isolate 2012EL-1759 might acquire *ctxAB* and give rise to a toxigenic clone that has a genomic backbone and accessory elements similar to those of the *V. cholerae* O1 classical biotype. The value of environmental surveillance and characterization is that we can compare them with outbreak isolates. If the environmental strain described here does give rise to a novel toxigenic strain in Haiti, we would properly be able to attribute its presence to emergence from the environment.

An article on the environmental sampling for this strain has been submitted for publication (Kahler et al., unpublished data).

### Nucleotide sequence accession numbers.

This whole-genome shotgun project has been deposited in DDBJ/ENA/GenBank under the accession no. JNEW00000000. The version described in this paper is the first version, JNEW01000000.
